# Long Non-coding RNA FIRRE Acts as a miR-520a-3p Sponge to Promote Gallbladder Cancer Progression via Mediating YOD1 Expression

**DOI:** 10.3389/fgene.2021.674653

**Published:** 2021-06-08

**Authors:** Shuqing Wang, Yang Wang, Shouhua Wang, Huanjun Tong, Zhaohui Tang, Jiandong Wang, Yongjie Zhang, Jingmin Ou, Zhiwei Quan

**Affiliations:** ^1^Department of General Surgery, Xinhua Hospital, Shanghai Jiao Tong University School of Medicine, Shanghai, China; ^2^Department of Hepatic Surgery, Eastern Hepatobiliary Surgery Hospital, Second Military Medical University, Shanghai, China

**Keywords:** lncRNA FIRRE, gallbladder cancer, cell proliferation, miR-520a-3p, YOD1

## Abstract

**Objectives:**

The role of lncRNAs in gallbladder cancer (GBC) remains poorly understood. In this study, we explored the function of functional intergenic repeating RNA element (FIRRE) in GBC.

**Materials and Methods:**

Whole transcriptome resequencing was performed in three pairs of GBC tissues and adjacent non-tumor tissues. lncRNA FIRRE expression was verified by real-time PCR. The function of FIRRE in GBC was evaluated by experiments *in vitro* and *in vivo*. The mechanism of FIRRE was investigated via fluorescent *in situ* hybridization, RNA pull-down, dual luciferase reporter assays, and RNA immunoprecipitation.

**Results:**

FIRRE level was dramatically increased in GBC tissues compared to that in the adjacent non-tumor tissues. High expression of FIRRE was closely related to clinical stage and poor prognosis in GBC patients. Moreover, FIRRE remarkably enhanced proliferation and migration, and inhibited apoptosis of GBC cells. Mechanistically, FIRRE modulated YOD1 expression by sponging miR-520a-3p, thus contributing to the development of GBC.

**Conclusion:**

Our data revealed that FIRRE might act as a novel mediator in GBC progression by sponging miR-520a-3p and regulating YOD1. FIRRE might be regarded as a potential diagnostic marker or target for GBC treatment.

## Introduction

Gallbladder cancer (GBC) is the most common malignancy in the biliary tract (Krell and Wei, 2019; Gu et al., 2020b). Lack of effective early diagnosis is one of the major causes of its poor prognosis. In the past few decades, despite improvements in exploratory surgical excision combined with chemotherapy or targeted therapy, the 5-year survival rate for operable locally advanced or node-positive gallbladder neoplasm is still unsatisfactory (Takada et al., 2002; Mayo et al., 2010; Witjes et al., 2012; Primrose et al., 2019). With the high speed development of cancer biology and gene sequencing technology, the molecular mechanisms of pathogenesis have been widely studied for GBC in recent years (Kanthan et al., 2015). Therefore, it is essential to identify clinically relevant biomarkers for diagnosis and treatment in GBC.

Long non-coding RNAs (lncRNAs) are a class of RNAs that are longer than 200 nucleotides in length, and they do not have the ability to encode proteins or peptides generally (Mercer et al., 2009). lncRNAs display various molecular functions through four mechanisms: signals, decoys, guides, and scaffolds (Wang and Chang, 2011). Emerging evidence demonstrated that dysregulated lncRNAs play vital roles in the development of tumors, including cell proliferation, migration, epithelial-to-mesenchymal transition (EMT), cell death, and chemoradiotherapy resistance (Chen et al., 2017; Peng et al., 2017, 2018; Liu et al., 2018; Xu et al., 2018). To date, a number of lncRNAs have been implicated in GBC. For example, lncRNA PVT1 could upregulate HK2 expression by sponging miR-143 to promote GBC progression (Chen et al., 2019). Our previous study revealed that the lncRNA MEG3 inhibits proliferation and invasion of GBC via increasing the ubiquitination of EZH2 (Jin et al., 2018). Therefore, it is urgent to identify novel tumor-associated lncRNAs and investigate their biological roles in order to discover novel approaches for early diagnosis and therapy of GBC.

In our present study, we revealed that an lncRNA, functional intergenic repeating RNA element, or FIRRE, acted as a cancer promoter of GBC. FIRRE was highly expressed and was closely related to poor prognosis in patients with GBC. Further function and mechanism studies showed that the lncRNA FIRRE enhanced cell proliferative and migratory capacity, and inhibited cell apoptosis via sponging miR-520a-3p to release microRNA for the target gene YOD1. Furthermore, we found that YOD1 executed its function of promoting GBC cell proliferation and migration, and inhibiting apoptosis. In summary, our studies revealed the FIRRE might provide feasible strategies for diagnosis and therapy against GBC.

## Materials and Methods

### Patient Tissue Samples and Whole-Genome Sequencing Analysis

A total of 60 GBC and 20 para-carcinoma tissue samples (including 20 pairs of samples) were collected from GBC patients who received surgery at Xinhua Hospital (Shanghai Jiao Tong University School of Medicine, Shanghai, China) and Eastern Hepatobiliary Surgery Hospital and Institute (Second Military Medical University, Shanghai, China) from 2013 to 2018. Three pairs of GBC and para-carcinoma tissues were subjected for whole-genome sequencing. All samples were stored in liquid nitrogen before RNA isolation (Shi et al., 2018a,b). The patients ranged in age from 49 to 84 years, with an average age of 68 years. All patients did not undergo any local or systemic therapy before the operation, and were staged based on the TNM classification system from the American Joint Committee on Cancer. Complete clinical and pathological data were obtained for all patients except for 11 who were lost to follow-up. Follow-ups after surgery were performed according to patient survival time until November 30, 2018. The clinical data of the patients are displayed in Supplementary Table 1. This study was supported by the Human Ethics Committee of Xinhua Hospital. All patients gave written informed consent.

### Cell Culture

In the present study, five human cell lines of GBC, including NOZ, GBC-SD, EHGB-1, SGC-996, and OCUG-1 were used. Moreover, a human intrahepatic bile duct epithelial cell line H69 was used. GBC-SD and OCUG-1 were obtained from the cell bank of the Chinese Academy of Sciences (Shanghai, China). NOZ cells were obtained from the Health Science Research Resources Bank (Osaka, Japan). EH-GB1 and SGC-996 cell lines were kindly supplied by the Eastern Hepatobiliary Surgery Hospital and Institute of the Second Military Medical University. The NOZ cell line was cultured in Williams’ medium E (Genom, China) containing 10% fetal bovine serum (FBS, Gibco, United States), and the remaining four GBC cell lines and H69 were cultured in DMEM high glucose medium (Gibco, United States) with 10% FBS. All cells were incubated at 37°C, 5% CO_2_ (Gu et al., 2020a).

### Real-Time PCR

Total RNA was isolated from tissues and cells by TRIzol reagent (Invitrogen, United States) according to the manufacturer’s protocol. RNA was reverse transcripted into cDNA using a Primer-Script One Step RTPCR kit (Takara, China). Real-time PCR was conducted with a SYBR Premix Dimming Eraser Kit (Takara, China). The primers were synthesized at the Shanghai Sanyuan Biotechnology Co., Ltd.; these are shown in Table 1. Results were standardized by the expression of β-actin. All experiments were repeated three times. The relative expression of all genes was analyzed using the 2^–ΔΔ*Ct*^ method.

**TABLE 1:** Information of the qRT-PCR primer.

FIRRE (Forward)	TGAAAGGGAATCCTGACGCC
FIRRE (Reverse)	TGCCTAGCTCTGACAATGGC
YOD1 (Forward)	ATGTTTGGCCCCGCTAAAGG
YOD1 (Reverse)	CGGTGATGGCGGCAATTTG
miR-520a-3p (Forward)	ACACTCCAGCTGGGAAAGTGCTTCCCTTTG
miR-520a-3p (Reverse)	TGGTGTCGTGGAGTCG
-actin (Forward)	AAAGACCTGTACGCCAACAC
-actin (Reverse)	GTCATACTCCTGCTTGCTGAT
U6 (Forward)	CTCGCTTCGGCAGCACA
U6 (Reverse)	AACGCTTCACGAATTTGCGT
**siRNA name**	**siRNA sequence (5′–3′)**
si-FIRRE-1 sense	CCAUGUACACCAUCAUCAATT
si-FIRRE-1 antisense	UUGAUGAUGGUGUACAAUGGTT
si-FIRRE-2 sense	GCCUAGGACCUUUGUGGUATT
si-FIRRE-2 antisense	UACCACAAAGGUCCUAGGCTT
si-YOD1 sense	UAAAACUUGGACAAAAUCGAU
si-YOD1 antisense	CGAUUUUGUCCAAGUUUUACC
si-Negative Control sense	UUCUCCGAACGUGUCACGUTT
si-Negative Control antisense	ACGUGACACGUUCGGAGAATT
**Probe name**	**sequence**
FIRRE	GACCACGCACAAACAGAUGAGAACCAAAA
	CCGAGUGAA
Control	CCAGTGAATCCGTAATCATG

### RNA Interference

Small interfering RNA (siRNA) and negative control (NC) sequences were synthesized via GenePharma (Shanghai, China). NC and siRNA fragments were transitorily transfected into cells using Lipofectamine 2000 (Invitrogen). After transfection, cells were incubated for 48 h. The siRNA sequences are shown in Supplementary Table 1. Real-time PCR was run to determine the knockdown efficiency.

### Cell Proliferation Assays

The proliferative potential of GBC cells was evaluated via Cell Counting Kit-8 (Takara, Dalian, China) and the Cell-Light^TM^ EdU DNA Cell Proliferation Kit (Ribobio, Guangzhou, China). In brief, cells transfected with si-FIRRE, si-YOD1, si-NC, miRNA mimic, or miRNA inhibitor were plated in 96-well plates (1 × 10^3^ cells/well). After 24, 48, 72, and 96 h, the absorbance at 450 nm was determined. GBC cells were fixed using 4% paraformaldehyde for 30 min, and stained by Hoechst 33342 (Beyotime, Shanghai, China) for 20 min. The stained cells were observed by a fluorescence microscope (Leica, Wetzlar, Germany). All the assays were repeated three times.

### Cell Cycle and Apoptosis Assays

After transfection by siRNA or si-NC and incubation for 48 h, cells were collected for cell cycle and apoptosis assays. In brief, for the cell cycle assay, cells were fixed for 16 h at 4°C, using pre-cooled 70% ethanol, and propidium iodide (PI) was used to stain the fixed cells. To measure cell apoptosis, we used an FITC-Annexin V Apoptosis Detection Kit (BD Biosciences), performed following the product instructions. Cell cycle and apoptosis data were detected via flow cytometry (Becton Dickinson FACSCalibur, NY, United States). All experiments were repeated three times.

### Wound Healing and Transwell Assays

After transfection for 24 h, the GBC cells were plated in a 6-well plate. When the cells covered approximately 70%, cells were scraped into the middle of the well using a 200-μL pipette tip. After 24 h of cell culture by serum-free medium, the wound widths of three independent wounds per group were checked. For the migration assay, a 24 transwell plate (Corning, United States) was used. The upper chamber was plated with 2 × 105 transfected cells, which were cultured by serum-free medium. The lower chamber was added with 500 μL of 10% FBS-containing medium. After 24 h of culture, cells that migrated into the bottom chamber were subject to fixation and staining, using 4% paraformaldehyde and 0.1% crystal violet, respectively. The stained cells were observed under a microscope (Leica, Wetzlar, Germany). Data were analyzed by five randomly selected fields for each sample.

### Western Blotting

The total protein was isolated by RIPA buffer (Beyotime, Shanghai, China). The concentrations of proteins were detected via a BCA kit (Applygen Technologies Inc., China). Proteins were separated on a 10% SDS-PAGE gel, and transferred to a PVDF membrane. After blocking via 5% skimmed milk powder, the membranes were probed with primary antibodies, including anti-YOD1, CDK4, CyclinD1, Bax, Bcl-2, Caspase- 3 (1: 1000; Cell Signaling Tech., Beverly, MA, United States), and anti-β-actin (1: 5000, Cell Signaling Tech.), at 4°C overnight. Then, secondary antibodies (1: 5000, Cell Signaling Tech.) were used to incubate the membranes at 20°C for 2 h. Protein bands were visualized by the enhanced chemiluminescence (Millipore, Billerica, MA, United States).

### Xenograft Mouse Model

This study was supported via the Animal Care and Use Committee of Xinhua Hospital. Stably expressing si-NC or si-FIRRE GBC cells (1 × 10^6^) were subcutaneously injected into the left sides of the flanking region of 3-week-old male nude mice, with five mice in each group. The volume of the tumor was determined (0.5 × length × width 2) and the tumor weight was monitored weekly. Four weeks later, the mice were sacrificed and the tumor tissues were collected.

### RNA Pull-Down Assay

Biotin-labeled probes for FIRRE and control were synthesized in Geneseed Biotech (Shanghai, China). After lysis, GBC-SD cell lysate was incubated using a FIRRE or control probe. Then the cell lysate was added with streptavidin-coated magnetic beads, which can bind to the biotin, thus pulling down the RNA complex. After removing the beads, the RNA was isolated from the product using TRIzol (Takara, Dalian, China). The enrichments of FIRRE and miR-520a-3p were evaluated via real-time PCR.

### Immunohistochemistry

The tumor tissues were subjected to fixing and embedding by 4% paraformaldehyde and paraffin, respectively. Followed by antigen retrieval and blocking, 3-μm tissue sections were probed by YOD1 antibodies (CST, United States) at 4°C overnight. After incubating by secondary antibodies (Beyotime, Shanghai, China) for 30 min, the sections were stained using diaminobenzidine. Each visual field was evaluated blindly under the light microscope by two pathologists.

### RNA Immunoprecipitation (RIP) Assay

The RIP assay was conducted by an EZ-Magna RIP^TM^ RNA-binding protein immunoprecipitation kit (Millipore, Billerica, MA, United States), following the procedure specification. In brief, a cell sample was lysed by the complete RIP lysis buffer (Millipore), which was supplemented with RNase and protease inhibitors. On the other hand, magnetic beads were pretreated with the specific antibodies. Especially, for the negative group, magnetic beads were added with anti-mouse IgG (Cell Signaling Tech.), and the experimental group was added with anti-AGO2 (Millipore). Then, the prepared magnetic beads were added with 100 μl of cell lysate to incubate the antibodies. The RNA was isolated from the immunoprecipitation product and evaluated by real-time PCR.

### Dual-Luciferase Reporter Assay

The whole length sequence of FIRRE and the 3′UTR sequence of YOD1, as well as their mutant sequences which mutate at the miR-520a-3p binding site, were synthesized, and cloned to the psiCHECK2 vectors (Promega, Madison, WI, United States). These wild-type and mutant-type vectors were named FIRRE-WT, FIRRE-Mut, YOD1 3′UTR-WT, and YOD1 3′UTR-Mut, respectively. The success of vector construction was verified via sequencing. The relative luciferase activities in each group were determined using a Dual-Luciferase Assay Kit (Promega, Madison, WI, United States), following the product specification.

### Statistical Analysis

The data were analyzed by SPSS 20.0 (SPSS, Chicago, IL, United States). Results were displayed as mean ± standard deviation (Pan et al., 2017; Sun et al., 2017). At least three biological replicates were performed for each group of experiments. Comparisons between two groups were analyzed by Student’s *t*-test. Kaplan–Meier survival analysis and log-rank test was used to analyze overall survival (OS) (Gu et al., 2017, 2018; Chen et al., 2020). It was considered significantly different when *P* < 0.05 (Gu et al., 2016).

## Results

### FIRRE Is Upregulated in GBC, and Its High Level Indicates Poor Prognosis in GBC Patients

**FIGURE 1:**
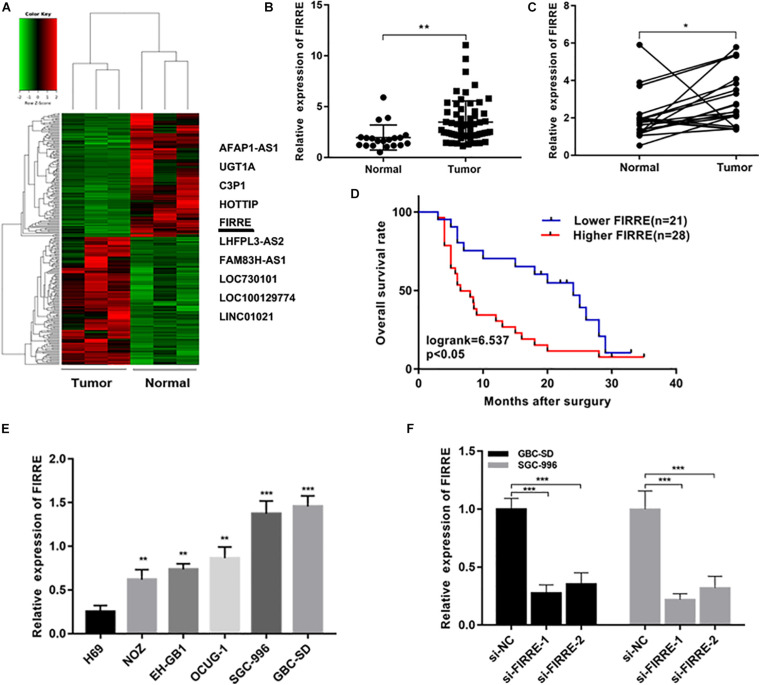
Relative expression of FIRRE in GBC tissues, cells, and its clinical significance. **(A)** The cluster heat map showed the differentially expressed lncRNAs in GBC tissues and adjacent non-tumor tissues. The top 10 upregulated lncRNAs were listed. **(B)** Relative expression of FIRRE in GBC tissues (*n* = 60) and non-tumor tissues (*n* = 20) was examined by qRT-PCR assays. **(C)** FIRRE expression levels in GBC tissues and paired neighboring non-tumor tissues (*n* = 20). **(D)** Kaplan–Meier analysis of overall survival according to FIRRE expression levels. **(E)** Relative expression of FIRRE in GBC cell lines and human biliary epithelium cell line H69. **(F)** Relative expression of FIRRE in GBC-SD cells and SGC-996 cells transfected with siRNAs. *p < 0.05, **p < 0.01, ***p < 0.001.

In order to understand the expression profiles of lncRNA in GBC, we applied whole-genome sequencing to obtain lncRNA expression profiles in three pairs of GBC and adjacent non-tumor tissue samples, and found that lncRNA FIRRE was abnormally elevated (Figure 1A). Subsequently, through the analysis of large samples by qRT-PCR, we further verified that FIRRE was significantly upregulated in GBC tissues compared to that in the adjacent non-cancer tissues (Figures 1B,C). Additionally, in order to find out the correlations between FIRRE level and clinical characteristics, 46 GBC samples were divided into two groups based on the FIRRE expressed median, including the low FIRRE group (*n* = 22) and the high FIRRE group (*n* = 24). Further statistical analysis revealed FIRRE overexpression was positively related to TNM stage. However, FIRRE level showed no significant relationship with age, gender, tumor size, histological grade, lymph node metastasis, and adjacent organ invasion (Supplementary Table 1). Interestingly, patients with highly expressed FIRRE showed a shorter survival time compared to those with lowly expressed FIRRE (Figure 1D). Using univariate survival analysis, we found tumor size, lymph node metastasis, high FIRRE level, and TNM stage were the factors influencing the prognosis of GBC patients. Multivariate analysis illustrated that the high level of FIRRE, as well as tumor size, were independent predictors that affected the prognosis of patients with GBC (Supplementary Table 1).

### FIRRE Facilitates GBC Cell Malignancy *In vitro*

**FIGURE 2:**
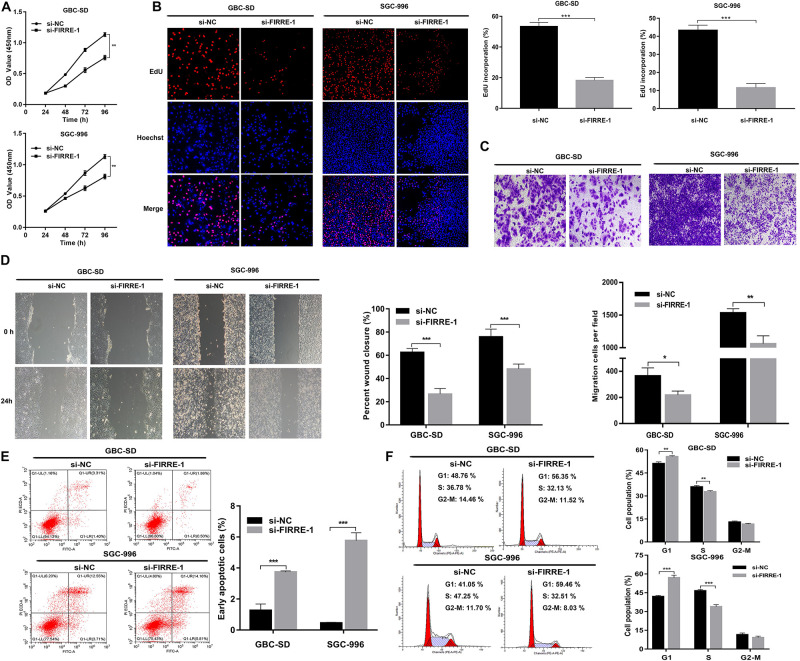
Effect of FIRRE on GBC cells proliferation, migration, cell cycle, and apoptosis *in vitro*. **(A)** The proliferation ability of GBC-SD cells and SGC-996 cells transfected with si-FIRRE-1 were determined by CCK-8 assays. **(B)** EdU assays were conducted in GBC cells after transfection with si-FIRRE-1 (magnification, ×100). Scale bar, 100 μm. **(C)** Cell migration was assessed in GBC cells in which FIRRE was inhibited. Scale bar = 100 μm. **(D)** The migration ability of transfected GBC-SD and SGC-996 cells by cell wound healing assays. **(E)** Apoptosis rate was analyzed by flow cytometry after downregulation of FIRRE. **(F)** Flow cytometric analyses were performed to determine the cell cycle progression in si-FIRRE-1-transfected GBC-SD cells and SGC-996 cells. *p < 0.05, **p < 0.01, ***p < 0.001.

**FIGURE 3:**
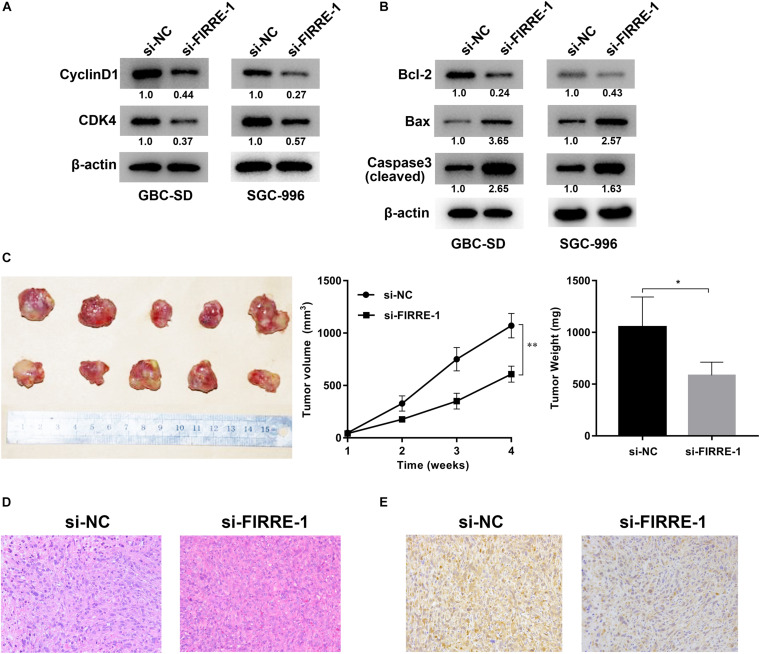
Effect of FIRRE on the proteins related to cell cycle and apoptosis, and xenograft tumor growth *in vivo*. **(A)** The expression levels of cell cycle-related proteins CyclinD1 and CDK4 were determined by western blot assays transfected with si-FIRRE-1 in GBC-SD and SGC-996 cells. **(B)** The expression levels of apoptosis-related proteins Bcl-2, Bax, and Cleaved caspase-3 in transfected GBC-SD cells and SGC-996 cells. **(C)** Representative example of nude mice at 4 weeks post-injection with subcutaneous xenografts of GBC-SD cells transfected with si-FIRRE-1 (five mice per group). Quantitative analysis of xenografted tumor volumes and weights. **(D)** HE staining of tumor tissues transfected with si-NC or si-FIRRE-1. **(E)** The YOD1 protein levels of xenograft tumors from si-NC or si-FIRRE-1 groups was determined by immunohistochemical staining. *p < 0.05, ^∗∗^*p* < 0.01.

We confirmed the FIRRE level in five human GBC cells (NOZ, GBC-SD, SGC-996, EH-GB1, OCUG-1) and a human normal biliary epithelial cell (H69) via real-time PCR. As expected, the expression of FIRRE was significantly upregulated in the GBC cells compared to the H69 cells (Figure 1E). To investigate the role of FIRRE, two siRNA fragments of FIRRE were transfected into GBC-SD and SGC-996, in which FIRRE was relatively highly expressed. Of the two siRNA fragments, si-FIRRE-1 showed a higher interference effect on GBC cells (Figure 1F). Therefore, si-FIRRE-1 was used for the subsequent studies. Subsequently, a CCK-8 assay was applied to evaluate the function of FIRRE in GBC cell growth. Results revealed FIRRE silencing remarkably suppressed the cell proliferative ability of GBC-SD and SGC-996 (Figure 2A). Consistently, the EdU assay showed FIRRE knockdown dramatically reduced the percentage of cells that were EdU positive (Figure 2B). Moreover, transwell and cell wound healing experiments were performed to determine the role of FIRRE in the migratory potential of GBC cell lines. We found that the migratory potential was markedly suppressed in FIRRE-downregulated GBC cells (Figures 2C,D). Subsequently, the action of FIRRE in the cell cycle and apoptosis was investigated in GBC cells. Flow cytometry analysis illuminated that si-FIRRE-1 significantly inhibited the cell apoptosis of GBC compared to si-NC (Figure 2E). With FIRRE silencing, GBC-SD and SGC-996 cells in the G0-G1 phase were significantly increased, while the cells in the S phase were decreased (Figure 2F), indicating that interference of FIRRE led to GBC cell arresting in the G1 phase. Additionally, the expression of proteins associated with the cell cycle and apoptosis was determined by western blotting. We found that when FIRRE was knocked down, the cycle-related protein of CDK4 and CyclinD1 as well as anti-apoptotic protein Bcl-2 were decreased in GBC cells (Figure 3A), while the proteins associated with pro-apoptosis, including Bax and cleaved caspase-3, were increased (Figure 3B). This evidence implied that FIRRE enhanced the development of GBC cells *in vitro*.

### FIRRE Facilitates GBC Tumorigenesis *in vivo*

To investigate the role of FIRRE in tumorigenesis *in vivo*, GBC-SD cells were transfected with si-NC or si-FIRRE, and then injected subcutaneously into nude mice. We found FIRRE deficiency significantly decreased the tumor sizes and weights, compared to the si-NC group (Figure 3C). In addition, HE staining of the tissue section found that knockdown of FIRRE exhibited a weak heterogeneity of the nucleus in the tumors compared to si-NC (Figure 3D). Finally, immunohistochemical staining found that downregulation of FIRRE could attenuate the protein level of YOD1 in xenograft tumor tissues (Figure 3E). Together, our data indicated that FIRRE might act as an oncogene in GBC.

### FIRRE Acts as the Sponge of miR-195-5p

**FIGURE 4:**
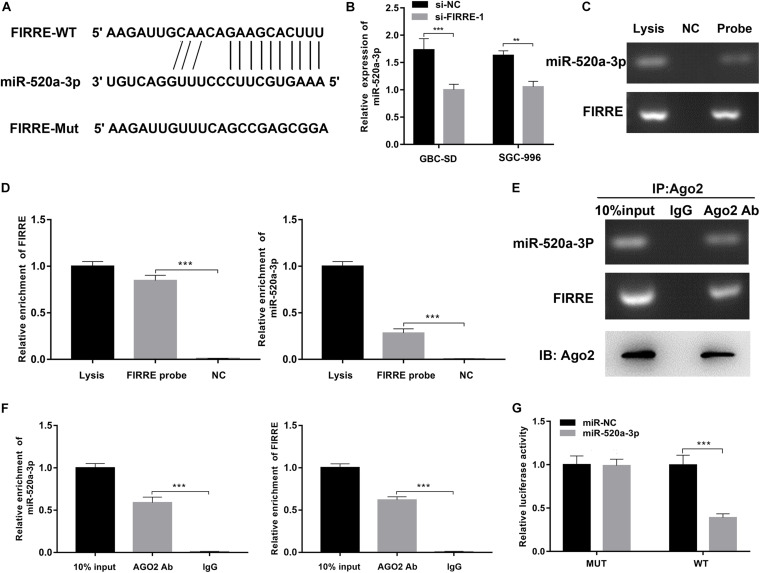
FIRRE functions as a sponge for miR-520a-3p. **(A)** The miR-520a-3p binding site on FIRRE predicted by miRanda and RNAhybrid. **(B)** The relative expression of miR-520a-3p was detected by qRT-PCR after transfection with si-FIRRE-1 in GBC-SD cells and SGC-996 cells. **(C,D)** RNA pull-down was executed in GBC-SD cells, followed by qRT-PCR to detect the enrichment of FIRRE and miR-520a-3p. **(E,F)** Anti-AGO2 RIP was executed in GBC-SD cells after transfection with miR-NC and miR-520a-3p mimic, followed by western blot and qRT-PCR to detect AGO2 protein, FIRRE, and miR-520a-3p, respectively. **(G)** The relative luciferase activities were detected in 293 T cells after transfection with FIRRE-WT or FIRRE-Mut and miR-NC and miR-520a-3p mimic, respectively. **p < 0.01, ***p < 0.001.

To clarify the regulatory mechanisms of FIRRE, first of all, target genes of FIRRE were predicted using the miRanda and RNAhybrid databases. We found FIRRE had a miR-520a-3p binding site, and the binding score of miR-520a-3p was higher than other miRNAs. To investigate the interactions between FIRRE and miR-520a-3p, we performed dual-luciferase reporter assays, via establishing two vectors that carry the WT or Mut FIRRE 3′-UTR sequences (Figure 4A). We observed that miR-520a-3p dramatically inhibited the luciferase activity of wild-type FIRRE. By contrast, inhibition was negative in the cells co-transfected with the Mut FIRRE 3′-UTR (Figure 4G), indicating that miR-520a-3p might inhibit the activity of FIRRE by directly binding FIRRE. Proverbially, miRNAs mediate the silencing of target genes via combining with Argonaute2 (AGO2), which is a core member in RNA-induced silencing complex (RISC). Therefore, to further better understand the interaction between FIRRE and miR-520a-3p, we performed RNA immunoprecipitation (RIP) assays using an AGO2 antibody in GBC-SD cells. Results revealed that AGO2 significantly enriched AGO2, FIRRE, and miR-520a-3p, compared to IgG (Figure 4C). As expected, we found that a high level of miR-520a-3p remarkably increased the amount of FIRRE pulled down by AGO2 (Figure 4D). To further verify the binding of FIRRE and miR-520a-3p, we performed an RNA pull-down assay with specific biotin-labeled FIRRE probes. Similarly, the expression of FIRRE and miR-520-3p was significantly increased in the FIRRE probe group compared to the control probe group (Figures 4E,F). Moreover, silencing of FIRRE observably elevated the levels of miR-520a-3p in GBC-SD and SGC-996 cells (Figure 4B). Together, these results suggest that FIRRE might function as an RNA sponge to suppress miR-520a-3p in GBC.

### FIRRE Effects GBC Cell Proliferation, Migration, and Apoptosis Through miR-520a-3p

**FIGURE 5:**
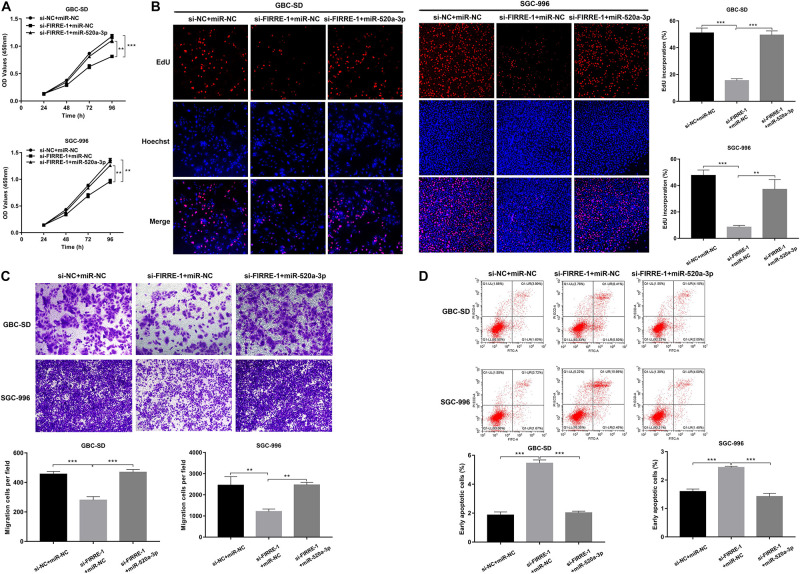
The proliferation, migration, and apoptosis in GBC cells co-transfected with si-FIRRE-1 and miR-520a-3p mimics. **(A)** The proliferation ability of GBC-SD cells and SGC-996 cells co-transfected with si-FIRRE-1 and miR-520a-3p mimics determined by CCK-8 assays. **(B)** The cell proliferation was determined by EdU assays. **(C)** The migration ability was examined after transfection with si-FIRRE-1 and miR-520a-3p mimics by transwell assays. **(D)** The apoptosis of co-transfected GBC-SD cells and SGC-996 cells. **p < 0.01, ***p < 0.001.

To further investigate the involvement of miR-520a-3p in the FIRRE-mediated tumor regulation process, we performed rescue experiments by miR-520a-3p mimics. Both CCK-8 and EdU experiments showed that silencing of FIRRE inhibited GBC cell proliferation, while miR-520a-3p mimics remarkably abrogated this effect (Figures 5A,B). Then, a transwell assay was performed to observe the migration affected by co-transfection with si-FIRRE-1 and miR-520a-3p mimics. The results indicated that the migration abilities were markedly promoted by miR-520a-3p mimics of the si-FIRRE group (Figure 5C). Furthermore, cell apoptosis was more reduced by co-transfection with si-FIRRE-1 and miR-520a-3p mimics than the si-FIRRE-1 group (Figure 5D). Together, our data indicate that FIRRE might contribute to the development of GBC by sponging miR-520a-3p.

### YOD1 Is Targeted by miR-520a-3p and Mediated by FIRRE

**FIGURE 6:**
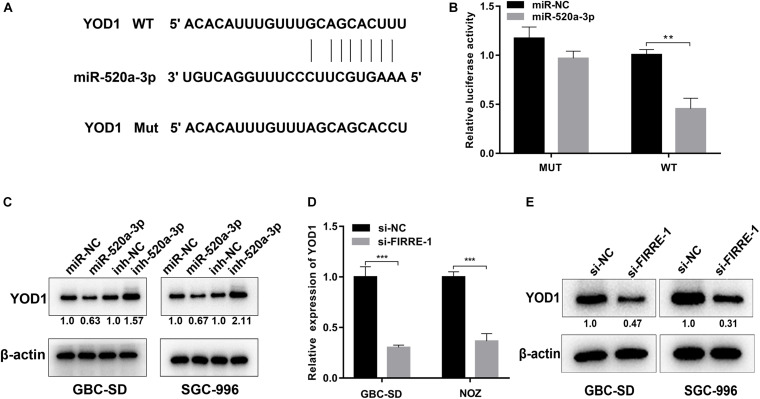
YOD1 is directly targeted by miR-520a-3p and indirectly regulated by FIRRE. **(A)** The 3′-UTR of YOD1 mRNA contains wild-type or mutant miR-30a-5p-binding sequences. **(B)** The relative luciferase activities were detected in 293 T cells after transfected with YOD1 3′UTR-WT or YOD1 3′UTR-Mut and miR-NC and miR-520a-3p, respectively. **(C)** Relative mRNA and protein levels of YOD1 were detected in GBC cells after transfection with miR-NC, miR-520-3p, inh-NC, and inh-520a-3p using qRT-PCR and western blot, respectively. **(D,E)** Relative expression levels of YOD1 was detected by qRT-PCR and western blot in cells transfected with si-NC or si-FIRRE-1. **P < 0.01, ***P < 0.001.

Using TargetScan (Gu et al., 2020c), we found that YOD1 and FIRRE had a common binding site in the miR-195-5p sequence. To verify the binding action of YOD1 and miR-520a-3p, we performed dual-luciferase reporter assays. As expected, miR-520a-3p remarkably inhibited the luciferase activity of wild-type YOD1, but had no effect on the mutant-type YOD1 (Figures 6A,B). Additionally, miR-520a-3p mimics remarkably suppressed the protein levels of YOD1, whereas the miR-520a-3p inhibitor remarkably increased the protein expression of YOD1 in GBC-SD and SGC-996 cells (Figure 6C). To validate whether FIRRE could regulate the expression of YOD1 in GBC cells, we found that knockdown of FIRRE markedly decreased the mRNA expression and protein level of YOD1 (Figures 6D,E). Collectively, the above evidence suggests that FIRRE mediates YOD1 via acting as a miR-520a-3p sponge in GBC.

### YOD1 Regulates GBC Cell Proliferation, Migration, and Apoptosis

**FIGURE 7:**
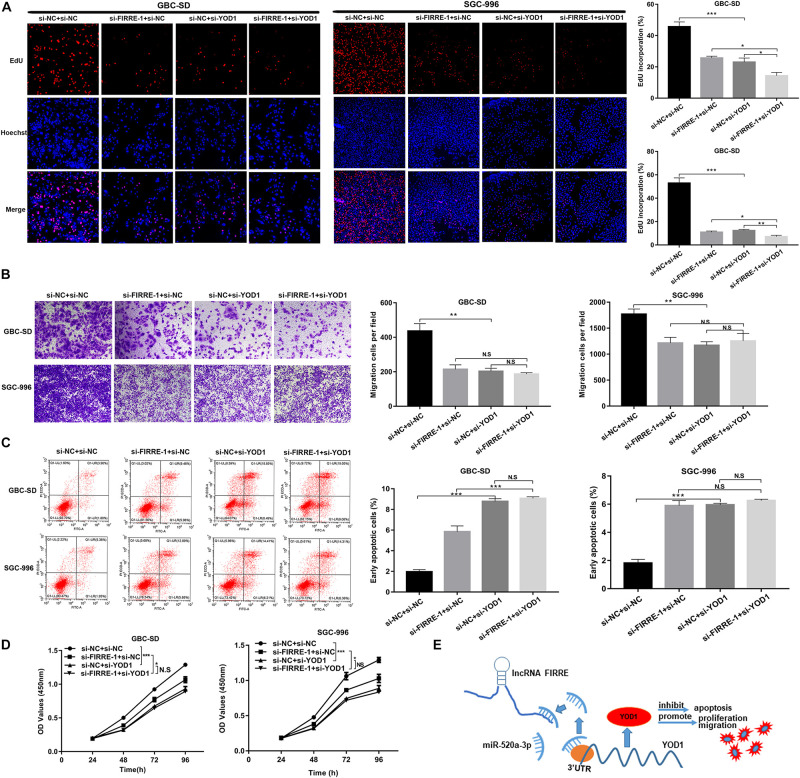
The proliferation, migration, and apoptosis in GBC cells co-transfected with si-FIRRE-1 and si-YOD1. **(A,D)** The proliferation ability of GBC-SD cells and SGC-996 cells co-transfected with si-FIRRE-1 and si-YOD1 were determined by CCK-8 and EdU assays. **(B)** The migration ability was examined by transwell assays. **(C)** The apoptosis of co-transfected GBC-SD cells and SGC-996 cells was analyzed by flow cytometric analyses. **(E)** Schematic diagram of how FIRRE promotes GBC tumorigenesis and progression. *p < 0.05, **p < 0.01, ***p < 0.001.

To investigate the function of YOD1 in the multiplication capacity of GBC cells, we carried out CCK-8 and EdU experiments. Interestingly, FIRRE or/and YOD1 knockdown significantly attenuated GBC cells proliferation (Figures 7A,D). Similarly, FIRRE or/and YOD1 downregulation decreased the migration ability in GBC-SD and SGC-996 cells (Figure 7B). To study the role of YOD1 in cell apoptosis, we also carried out flow cytometry assays. As expected, we observed that YOD1 induced the apoptosis of GBC cells (Figure 7C). Together, our results show that FIRRE might modulate YOD1 by serving as a sponge of miR-520a-3p, resulting in the occurrence and development of GBC (Figure 7E).

## Discussion

Emerging evidence has revealed that lncRNAs play vital roles in the occurrence and development of various diseases, especially the malignant development of a tumor (Chi et al., 2019). Although alterations of multiple lncRNAs during the development of GBC have been widely investigated (Wang et al., 2016; Cai et al., 2017), the functions and regulatory mechanisms of numerous GBC-related lncRNAs are still largely unknown.

Here, we applied whole-genome sequencing to obtain lncRNA expression profiles in three pairs of GBC and adjacent tissue samples, and observed that lncRNA FIRRE was abnormally elevated. Subsequently, we further verified that it was dramatically increased in GBC tissues and cell lines, and was significantly related to lymph node metastasis, pathological typing, clinical stage, and overall survival. In addition, functional experiments *in vivo* and *in vitro* revealed that silencing of lncRNA FIRRE dramatically reduced the proliferation and migration capacity of GBC cells, while increasing apoptosis. The full name of FIRRE is functional intergenic repeating RNA element, which is a newly identified lncRNA and localizes on the X chromosome. Previous research shows that lncRNA FIRRE anchors the inactive X chromosome via sustaining H3K27me3 methylation (Yang et al., 2015). Recent research demonstrated that FIRRE modulates nuclear architecture across chromosomes and interacts with the nuclear matrix factor hnRNPU (Hacisuleyman et al., 2014). lncRNA FIRRE enhances cell proliferation and inhibits cell apoptosis of DLBCL by inducing the Wnt/b-catenin signaling pathway (Shi et al., 2019).

The ceRNA mechanism is one of the approaches that lncRNA is involved in cell regulation which was first proposed by Salmena et al. (2011). In this hypothesis, any RNA molecule with a microRNA response element (MRE) site can release miRNAs with similar MRE from acting on downstream target molecules (Salmena et al., 2011). For example, LINC00152 positively modulates HIF-1a by binding miR-138 to promote metastasis and EMT in gallbladder cancer (Cai et al., 2017). lncRNA MT1JP can regulate the development of gastric cancer via modulating FBXW7 levels through serving as a ceRNA and competitively binding to miR-92a-3p to Zhang et al. (2018). In the present study, bioinformatics analysis revealed that FIRRE contains miR-520a-3p MRE. Furthermore, we confirmed that FIRRE can directly interact with it and verified this by dual-luciferase report, AGO2-RIP, and RNA pull-down analysis. Similarly, previous evidence also showed that miR-520a-3p has different degrees of change in different tumor tissues (Liu et al., 2016; Li et al., 2017; Bi et al., 2019). Bioinformatics analysis, WB experiments, and dual-luciferase report analysis confirmed that YOD was targeted and regulated by miR-520a-3p.

YOD1 is a highly conserved deubiquitinating enzyme belonging to the ovarian tumor (otubain) family, whereas its role and molecular mechanism remain unclear in mammalian cells (Ernst et al., 2009). A previous study showed that high expression of YOD1 can promote cell migration via triggering the TGF-β3 pathway, thus playing a vital role in lip and palate formation. Moreover, mutation of YOD1 can lead to abnormal TGF-β3 signaling, which inhibits the cell migration in NSCLP (Ju et al., 2018). YOD1 is involved in the modulation of endoplasmic reticulum (ER)-related degeneration pathways (Rumpf and Jentsch, 2006). In fact, YOD1 has been revealed to play important roles in the endoplasmic reticulum stress response triggered via the mis-localization of unfolded proteins in mammalian cells (Ernst et al., 2009; Claessen et al., 2010; Bernardi et al., 2013; Sasset et al., 2015). Emerging evidence showed that YOD1 functions as an important modulator of the inflammatory cytokine interleukin-1 via directly binding to TRAF6 (Schimmack et al., 2017). YOD1 reduces the aggregation of MAVS by the de-ubiquitination of K63, thereby inhibiting the body’s innate immune response (Liu et al., 2019). In this study, we demonstrated that FIRRE knockdown led to a correspondingly reduced YOD1 protein level, which in turn led to a reduction in the growth of GBC. However, which YOD1 pathway or signaling pathway influences the development of GBC needs further study. Previous studies have confirmed that YOD1 is a key modulator for the hippo signaling pathway. YOD1 clears the itching ubiquitin, enhances its stability, and induces LATS deterioration and YAP/TAZ activity. At the same time, the induced expression of YOD1 in the liver enhanced liver cell proliferation and caused liver hypertrophy in a YAP/TAZ activity-dependent manner (Kim and Jho, 2017; Kim et al., 2017).

There are some limitations in our study. First, the number of samples is still limited and needs further validation; second, the samples used for whole transcriptome resequencing are also limited and more bioinformatics methods should be performed to predict the ceRNA network.

## Conclusion

This study reported for the first time that lncRNA FIRRE was upregulated in GBC tissues and cells, and its high level might be a factor in the poor prognosis of GBC patients. In addition, FIRRE regulates YOD1 by sponging miR-520a-3p to promote cell proliferation and migration, and inhibit apoptosis of GBC. Our findings suggest that FIRRE may be helpful for lncRNA-guided GBC diagnosis and treatment.

## Data Availability Statement

The original contributions presented in the study are included in the article/Supplementary Material, further inquiries can be directed to the corresponding authors.

## Ethics Statement

The studies involving human participants were reviewed and approved by the Human Ethics Committee of Xinhua Hospital. The patients/participants provided their written informed consent to participate in this study.

## Author Contributions

ShuW and YW performed the experiments, collected and analyzed the data, and wrote the manuscript. ShoW and HT performed some experiments and provided technical and material support. ZT, JW, and YZ provided the clinical specimens of GBC. JO and ZQ contributed to the conception of the study, and designed and organized the study. All authors contributed to the article and approved the submitted version.

## Conflict of Interest

The authors declare that the research was conducted in the absence of any commercial or financial relationships that could be construed as a potential conflict of interest.
